# Direct-acting Antiviral in the Treatment of Chronic Hepatitis C: Bonuses and Challenges

**DOI:** 10.7150/ijms.43079

**Published:** 2020-03-15

**Authors:** Haiyan Zeng, Lei Li, Zhouhua Hou, Yapeng Zhang, Zhongxiang Tang, Shuiping Liu

**Affiliations:** 1 Department of Infectious Disease, Xiangya Hospital, Central South University, Changsha 410008, China; 2 Department of Microbiology, School of Basic Medical Science, Central South University, Changsha 410078, China

**Keywords:** hepatitis C virus (HCV), direct acting antiviral (DAA), sustained virological response (SVR), resistance-associated substitutions (RAS)

## Abstract

Owing to the rapid development and wide clinical application of direct acting antiviral (DAA) drugs in the treatment of hepatitis C virus (HCV) infection, the era of interferon-based therapy has almost come to an end. Cumulative studies show that DAA therapy renders high cure efficiency (>90%) and good safety profile, and may even bring some unexpected benefits to the patients. However, some issues of concern arise, one of which is the resistance mutation of HCV genome leading to failure of treatment. With the aim of providing some meaningful references for the treatment of chronic hepatitis C (CHC), this article summarizes the research progress on benefits of DAA accompanied by viral clearance in the treatment of chronic hepatitis and the drug resistance.

## Introduction

Hepatitis C virus (HCV) infection is a global epidemic. According to the World Health Organization (WHO), approximately 71 million people are currently living with hepatitis C virus worldwide, which is the main cause of chronic hepatitis. The number of death from hepatitis C-related liver disease has exceeded 399 000 yearly 1. Nowadays, it is well recognized that chronic hepatitis C (CHC) is a major public health problem that can contribute to liver fibrosis, progressive decline in liver function, and ultimately death in patients 1, 2.

As an infectious agent with high sequence variations, HCV is classified into seven genotypes (GT) and is further subdivided into nearly 100 subtypes to date. It is recognized that HCV genotypes have a regionally distinct global distribution: HCV GT1 (46.2%) and 3 (30.1%) dominate the global infections; GT 2, 4, and 6 are responsible for approximately 22.8% of HCV infections; GT 5 accounts for the remaining less than 1%; GT 7 has been identified so far in very few patients originating from Central Africa 3.

In the past two decades, the only standard treatment for patients with HCV infection is peg-interferon plus ribavirin (PegIFN/RBV) in 24 to 72 weeks, but only a limited proportion of patients can achieve a sustained virological response (SVR), defined as HCV RNA continues to be undetectable in serum at 12 weeks after completing treatment. What is worse, the interferon-based agent can cause many adverse effects in more than 10% of patients during and after the treatment 4, such as depression, cytoreduction and hemolytic anemia 5. Fortunately, as DAA drugs have been developed rapidly and used in the clinical application, the cure rate of chronic hepatitis C has a revolutionary improvement even in patients with liver cirrhosis, and the efficiency persists to rise from the first generation to the third generation DAA drugs 6.

In general, DAAs can be divided into three major classes based on their targets in HCV proteins: the nonstructural protein 3/4A (NS3/4A) protease inhibitors (PIs) that can inhibit HCV polyprotein processing; NS5A inhibitors, inhibiting viral replication and assembly; and NS5B polymerase inhibitors that can block HCV RNA replication 7. Specifically, each class of DAA includes several different clinical drugs, and nowadays two or three DAA combination therapies are recommended and clinically adopted.

Moreover, DAA combination therapies can achieve high SVR regardless of the HCV GTs and subtypes, even in patients with compensatory cirrhosis and decompensated liver disease.

Surprisingly, numerous studies have shown that DAA can not only effectively and safely remove HCV, but also achieve some unexpected benefits when compared with interferon-based therapy, such as repairing liver function damage, recovery of metabolic impairment and restoration of immunity dysfunction , etc. caused by HCV infection, and the number of patients with adverse effects are much lower. However, despite the excellent antiviral potency and good compatibility of DAA therapy, the consequent challenges such as the association between DAA regimen and tumor genesis 8, 9, and drug resistance which is the main leading factor to the failure of DAA treatments have already attracted the attention of many researchers. Therefore, this article focuses on the research progress of the extra performances of DAA and its drug resistance.

## Liver Function Repair

In HCV-infected people, only a minority of those can spontaneously recover from HCV infection, relying on their strong immunity. More than 70% of people will gradually develop chronic hepatitis C virus infection, and a considerable proportion of patients eventually develop into liver fibrosis, cirrhosis, *etc.* 10-12.

Liver function of more than two-thirds of patients will be damaged by HCV infection. Clinically, chronic HCV infection will contribute to obvious change of some related parameters: high concentration of transaminases (alanine aminotransferase and aspartate aminotransferase), elevated bilirubin concentration, elevated serum globulin concentration, albumin concentration, and lower platelet count. In addition, liver stiffness values and Child-Pugh grading standards can be used to measure the severity of the related liver disease. These data have a positive or negative correlation with the severity of liver function damage.

In recent years, mounting clinical trials have manifested that successful DAA treatment can not only effectively remove HCV, but simultaneously repair liver function damage due to HCV infection in at least two-thirds of the patients 13. It appears that the parameters related to liver function damage gradually approach the normal interval at the end of treatment and after a long period of time. For example, Edoardo G. Giannini *et al*. have observed significant change of related parameters, which demonstrated improvement of liver function in a prospective study. In the study, they observed the evidently dropped aminotransferases (*P* < 0.0001), a progressive increase in serum albumin (*P* = 0.010), and a decrease in serum bilirubin (*P* = 0.011) as well as in gamma globulin (*P* = 0.0003) between baseline level and that after treatment 14. A similar change was observed in other studies related to DAA and liver function repair 15, 16, which confirms this tendency.

Particularly, there are some studies that have evaluated global prognostic scores for patients with decompensated liver disease, such as the end-stage liver disease model (MELD), showing that the liver function of about 50% of patients has been improved after HCV was removed by DAA. Additionally, these improvements of liver function have been made in the short term 13, 17. At the same time, some studies have also shown that portal hypertension has also improved in subjects with successful DAA therapy, which has gradually become an independent predictor of hepatocellular carcinoma 13. Therefore, to some extent, DAA treatment can greatly reduce the incidence and relapse of hepatocellular carcinoma 18, 19.

Interestingly, with respect to patients who were diagnosed as liver fibrosis and cirrhosis, R. Flisiak *et al.* found that their liver function improvement after DAA treatment was often better than that of non-cirrhotic patients 15. The success of DAA treatment can reduce the liver stiffness value, which means the regression of fibrosis, the down-regulation of inflammatory activity and the improvement of blood circulation and the regression of hepatic steatosis 20. In another study, Edoardo G. Giannini *et al*. also revealed that at least 85% of patients with cirrhosis had a decrease in liver stiffness that dropped by about 40%. However, there were still 10% of patients who had liver stiffness values enhanced, although the virus has been eradicated after DAA treatment 17.

In view that most of these conclusions are based on the fact that the patient is mono-infected with HCV, we wonder whether the DAA treatment has the same effect of clearing HCV and repairing liver function damage for human immunodeficiency virus (HIV)/HCV co-infected patients, because it is well accepted that the HIV/HCV co-infection greatly increases the risk of developing advanced liver disease 21-23, and accelerates liver fibrosis in patients with chronic hepatitis C^24^.First of all, Cure rates of over 90%, similar to those in HCV mono-infected patients, can now be achieved in HIV/HCV co-infected patients. This has been documented in clinical trials 25-27 as well as in real-life cohorts 28-30. Furthermore, there are also studies showing that DAA therapies can achieves liver function repair in patients with co-infection. For instance, a study by Juan Macías *et al.* demonstrated that DAA treatment also improved liver function impairment in co-infected patients, and the degree of improvement in liver function was similar to that of patients with HCV mono-infection 31. After multivariate analysis, they showed that only baseline levels of serum albumin and overall deterioration of liver function are independently associated in HIV/HCV co-infected patients; in other words, there is no significant correlation between the status of HIV and the damage and repair of liver function. Consequently, it was concluded that patients with successful DAA treatment, regardless of whether HIV/HCV co-infection or HCV mono-infection, can obtain similar recovery of liver function.

## Recovery of Metabolic Damage

The association between HCV infection and dysregulation of metabolic processes has been observed since long ago. Furthermore, chronic HCV infection exerts a significant impact on the development of heart disease and stroke 32. Increasing epidemiological studies have long demonstrated that the prevalence of type 2 diabetes mellitus (T2DM) is much higher in subjects with chronic hepatitis C (CHC) than in the general population, ranging between 13% and 67% according to liver fibrosis stage and time of infection 33-35.

The hypothesis that HCV has a direct and important role in the regulation of glucose metabolism is supported by laboratorial investigations. Kasai D *et al*. showed that HCV replication can down- regulate the glucose transporter 2 expressions, which located on the surface of the cell, thereby affecting cellular uptake of glucose 36. Deng L *et al*. found that HCV up-regulated hepatic glucose production via NS5A-mediated FoxO1-dependent pathway 37. Recently, more systematic mechanisms underlying disorders of glucose metabolism caused by HCV infection have been observed in many experimental and clinical studies. HCV may directly inhibit the insulin-signaling pathway, with downregulation of glucose transporter 2, promotion of IRS-1 degradation through protein kinase B (Akt)/mammalian target of rapamycin (mTOR) activation, and suppression of phosphorylation of tyrosine on IRS-1. Moreover, HCV impairs phosphorylation of Akt, leading to a reduction in insulin stimulation 32.

The complex association between HCV infection and dysregulation of lipid metabolism has also been observed in recent years. Numerous studies have shown that HCV is highly dependent on the host's lipid metabolism to create an environment more suitable for its replication. HCV associates with lipoproteins to form a structure called lipoviral particle (LPV), released by hepatocytes 38-40, which in turn facilitates HCV evading from host immune responses and infecting new cells 40. Lipid virions are very similar to very low-density lipoproteins (VLDL), not only by the apolipoprotein content, but also by the lipid composition and particle density 38-41. Likewise, VLDL secretion pathway of infected cell is redirected by the virus especially for virion maturation/secretion, and the low-density lipoprotein (LDL) receptor is an essential component employed by HCV during cell adsorption and entry 42. Therefore, in HCV-infected patients, lipid metabolism is often observed to be down-regulated, which is characterized by decreased serum lipoprotein and total cholesterol, and substantial lipid accumulation 39.

Because of the serious metabolic disorder and consecutive related diseases due to HCV infection, the question of whether the success of DAA treatment can repair the metabolic damage arises. Until now, progressing studies have shown that DAA treatment has some beneficial impacts on both glucose metabolism and lipid metabolism.

Several studies indicated that DAA therapies indeed change the state of glucose metabolism in patients with the clearance of HCV. However, there is a certain contradiction in the results of these studies. A retrospective study carried out on 300 patients suggested that blood glucose levels significantly decreased in subjects with diabetes who achieved SVR. And most of the observed variation occurred early in time and in temporal concert with HCV clearance. Furthermore, the metabolic improvement was persistent with a reduction of average fasting glucose level for >1 year after the end of therapy 32.Consistent with the conclusion, there are some studies that reported similar results; a reduction of fasting glucose levels after HCV clearance had no obvious association with HCV genotype, body mass index (BMI) of patients and the DAA regimen used 43, 44.

In contrast, Philip Weidner *et al.* discovered that SVR is closely associated with the decline of fasting blood glucose levels in patients, and this decline will continue until 24 weeks after the completion of the treatment. However, the fasting blood glucose concentration will return to baseline level after 48 weeks 45. In addition, a 5-year's follow-up study by Jia Li *et al.* carried out on 384 HCV patients with T2D revealed that there was no significant difference in the concentration of HbA1c between untreated patients and those with treatment failure, whereas the variation of concentration of HbA1c in patients with successful DAA treatment experienced three stages: average HbA1c started off at roughly 7.7 and decreased significantly over time (*P*<0.001); then the concentration of HbA1c began to rise slowly (*P*=0.003); finally, HbA1c stabilized at an average level of 7.9 (p-value for the slope=0.337) 46.

The contradictory of these results may be explained by the length of follow-up after the end of treatment as well as different characteristics of subjects employed. In summary, a conclusion can be drawn that DAA have a positive impact on glucose metabolism damage early in time after the completion of DAA treatment, but this beneficial effect will gradually disappear as the follow-up time is extended.

With respect to lipid metabolism impairment in patients, unlike the short-term beneficial impact of DAA therapies on glucose metabolism, numerous studies have supported the hypothesis that HCV clearance after therapy is associated with a significant improvement in the lipid profile. A study by Gilmar de Souza Lacerda *et al*. found that the level of serum total cholesterol (*P*<0.0001), low density lipid protein- C (*P*<0.0001), VLDL-C (*P*=0.0003) and triglyceride (*P*=0.0003) were greatly elevated in patients who had acquired SVR at the end of treatment and 1 year after the end of treatment, while HDL-C levels in serum were not significantly changed, comparing with the baseline level 47. Similar results were observed in the study by Matt Driedger *et al.* They found out that post-treatment levels of cholesterol (n=36) and triglycerides (n=28) were also evaluated. Overall, a numerical increase was observed from baseline in both total cholesterol (*P*=0.06) and triglyceride levels (*P*=0.40). And patients with diabetes were noted to have an elevation in cholesterol after treatment (*P*=0.05 by Wilcoxon test; n=10), but no significant change in triglycerides (*P*=0.50 by Wilcoxon test; n=7) 48.In line with these results, several clinical studies provided strong evidence that lipoprotein levels and liver lipid accumulation were inclined to normalize after the initiation of combination therapy including sofosbuvir/ribavirin, sofosbuvir/ledipasvir, asunaprevir/daclatasvir and grazoprevir/elbasvir. These modifications appear to be associated with significant improvement of steatosis and atherogenesis 49-53.

Accordingly, these results support the conclusion that lipid metabolism damage caused by HCV can be completely reversed after DAA successfully removes the virus, which can improve the liver lipid degeneration and atherosclerosis 32.

To conclude, the question of whether DAA treatment can exert a beneficial impact on the metabolic impairment can be answered to date: DAA indeed can recover lipid metabolism damage, but only a short-term positive outcome on glucose metabolism damage.

## Rehabilitation of Immune Damage

A great deal of studies have shown that HCV infection will induce the up-regulation of many genes involved in innate immunity characterized by up-regulation of interferon-stimulated genes (ISGs) expression 54-56, elevated levels of interferon- sensitive cytokines and chemokines 53, 57-60. More importantly, the chronic activation of the innate immune response and the consequent activation of hepatic stellate cells are the initiators of hepatitis and cirrhosis 61. HCV may interact with immunity response through multiple mechanisms. It is well known that HCV RNA can be recognized by the Toll-like receptor 3 or the RIG-I helicase-mediated pathway in the cytoplasm, resulting in transcriptional activation of type 1 interferon. And type 1 interferon can activate the JAK-STAT signal pathway, which subsequently precedes the transcription of ISGs that have antiviral effects 62. Simultaneously, the increased type 1 IFN can also trigger natural killer cells and make it a polarized phenotype, elevated cytotoxicity, and down-regulation of the pro- apoptotic factors TRAIL and cytokines 63.

Moreover, in addition to changing innate immune response, HCV infection also leads to changes in specific immune response. In patients with chronic HCV infection, T cell responses can only be detected at low levels 64. This is because sustained antigenic stimulation results in up-regulation of T cell depletion markers, such as programmed cell death protein 1 (PD-1), programmed death-ligand 1 (PD-L1), T-cell immunoglobulin and mucin-domain containing-3 (Tim-3),cytotoxic T lymphocyte antigen 4(CTLA-4),CD160, B- and T-lymphocyte attenuator 65, which indicates the increasing population of phenotype of depleted T cells. Ultimately, the outcome of changes in the expression of these genes is that, partial or total loss of antiviral function and proliferation of T cells. This result can also be verified in animal models that the population of depleted T cell was found to be attenuated after IFN signaling 66.

Unlike interferon-based treatments, DAA treatment acts precisely on some critical steps of HCV replication, thereby preventing HCV replication. It plays a role in the treatment of CHC less dependent on the host's immune function. Hence, many researchers speculate that the dysfunction of CHC patient's immune system may be recovered partially or completely with the DAA clearing HCV.

To date, there is an extensive body of evidence that innate immune and specific immune response damage can be recovered in subjects with HCV clearance by DAA treatment, and this restoration presents long-term effect. According to a study of Matthew A. Burchill *et al.*, interferon-free DAA treatment can trigger a global rearrangement of innate immune signals and inflammatory pathways in patients with chronic hepatitis C. They observed an obvious reduction in the transcription of the cytokine IL1β involved in innate immune activation, hepatic inflammation, and fibrosis parallel with the deregulated phosphorylation levels of NF-ΚB protein that is associated with the activation of downstream signaling and innate immune. In addition, the level of C-X-C motif chemokine (CXCL)-10 and CXCL 11 also decline rapidly during treatment acting as chemokines, which can guide the innate immune cells to accumulate in the inflammatory part. This phenomenon indicates a reduction of the levels of HCV RNA in peripheral blood and an amelioration of liver inflammation. In addition, the expression of innate immune signaling related molecules such as retinoic acid induced gene I (RIG-I), Signal transducing activator of transcription 1 (STAT1), and interferon regulatory factor 7 (IRF7) is down- regulated, therefore, the rapid dampening of innate immune activation following rapid viral clearance with IFN-free DAA therapy is independent of the treatment regimen utilized^67^, ^68^.

In fact, the repair of immune dysfunction stemmed from DAA therapies changes the expression of major immune-related molecules, and the number and phenotype of innate immune cells and specific immune cells are also changed. Eric G. Meissner *et al.* found out that the concentration of lymphocyte in peripheral blood was increased dramatically during the first two weeks of DAA treatment, but this upregulation did not last for the entire treatment process. The increase of the population of T lymphocytes in peripheral blood can be reflected as a combined effect of reduced intrahepatic migration of lymphocytes due to changes in chemotaxis and potential outflow of intrahepatic lymphocytes 69. The most obvious visual variation over the course of treatment was a reduction in CD8 signal in the parenchymal and nonparenchymal regions of liver, which was observed in all patients who received DAA treatment, as well as some patients with treatment failure. Hence, this result indicates that the frequencies of CD8+T cells may have no correlation with the treatment outcome. Similarly, in clinical trials, Cody Orr *et al*. speculated that the decrease of population of CD8+ T cells may be due to a decrease in HCV concentration, contributing to an intrahepatic cellular response to reduction in viral burden. In addition, the population of CD4+T cells also declined in the non-parenchymal region of liver, while the significant variation of frequencies of Kupffer cells associated with inflammation was not observed 70.

Furthermore, studies by Shikha Shrivastava *et al*. showed that the expression of depleted T cell markers was reduced during DAA treatment, and the proportion of exhausted phenotypes T cell was obviously declined ,but the proportion of HCV-specific CD8+ cells is correspondingly heightened. Because the exhausted T cells partially or completely lose their ability to secrete antiviral cytokines such as IFN-α, IFN-γ, and IL-2. However, after successful treatment, compared with baseline levels, the expression levels of these cytokines are increased in T cells, which mean a recovery to some extent in HCV-specific immune response. Thence, amplification in HCV-specific CD8+ T cell responses is a direct result of decreased expression of T cell depletion markers 71. In addition, 4 years after HCV clearance by DAA, a steady growth in the number of regulatory T cells was observed in patients, indicating that DAA has a sustained effect on immune function in the liver for a long period of time after the clearance of HCV 72. Interestingly, however, the memory phenotype of CD4 T cells and CD8 T cells did not make changes during the course of treatment.

Additionally, it is well documented that patients co-infected with HIV and HCV have higher levels of immune activation and impaired antigen-specific responses compared to patients mono-infected with HCV^65^.It is important to assess the impact of DAA therapies on recovery of immune dysfunction in subjects with HIV/HCV co-infection. Several small studies have demonstrated improvement in liver ISG expression, restoration of type I IFN signaling, and natural killer and T cell function following IFN-free DAA therapy in the setting of chronic infection 73-75.Furthermore, DAA combination therapies in patients co-infected with HIV/HCV resulted in similar restoration of the T-cell impairments and perturbations associated with HIV/HCV coinfection to an extent. However, the effect of improvement of immune response in patients with DAA treatment is different, as the result of Shikha Shrivastava *et al*. shown that patients co-infection with HIV/HCV lead to greater restoration of the immunologic outcomes to an extent that was greater in three-drug combination therapies than that observed in either two-drug regimens^65^.Consequently, a conclusion can be drawn that successful DAA treatment indeed restore the dysfunction of immunity system to some extent in patients with HIV/HCV co-infection.

Thus, available results all favored the conclusion that immune impairment due to HCV infection and HCV/HIV co-infection after the end of successful DAA treatment, which is characterized by the reduction in proportion of exhausted T cells and enhancement of virus-specific T cells response, and remarkable amelioration of liver inflammation, can be observed in the responders. Furthermore, lacking continuous IFN stimulation in the liver after clearance of the virus with DAA will likely also have a significantly beneficial effect on intrahepatic immune responses.

## Drug Resistance

Although DAA presents a high SVR for HCV-infected patients with the six major genotypes (HCV GT3 patients have a slightly lower SVR), it still confronts with the challenge of drug resistance, especially resistance-associated substitutions (RASs), which is the main reason of treatment failure. Drug resistance is an intrinsic and unavoidable problem in antiviral therapy because of the high adaptability of HCV and the failure to maintain a high pressure of inhibition 77, and the emergence of RASs will reduce the cure rate of the drug.

It is well known that resistance mutations are favored by the lack of efficient proof-reading activity of HCV RNA-dependent RNA polymerase, which elevated to be between 10^3^ and 10^5^ per copied base pair, as well as the high rate of viral replication (up to 10^12^ particles produced per day), leading to one mutation for every genome copied. As a consequence, HCV exists as a quasi-species population: a complex mixture of genetically distinct but closely related viral populations that constitutes a reservoir for the emergence of resistant strains 78. Not surprisingly, resistance emerges when replication occurs in the presence of drug-selection pressure. The probability of a mutation associated with drug resistance being selected out during therapy relies on the potency of that drug. Undoubtedly, in addition to the RASs emerging after virological failure, there are some resistance mutations that occur naturally before treatment, which both have a negative effect on cure rate, and may eventually resulting in treatment failure. In general, there are many factors associated with the occurrence of RASs, including therapy regimen, genotype and subtype of HCV and geographical distribution.

Encouraging clinical and retrospective study have shown that the prevalence of RASs in the gene regions of three non-structural proteins of HCV is not the same, and this may be due to their different roles in HCV life cycle, which determines the resistance barriers of the three non-structural proteins of HCV are different (the genetic barrier to drug resistance defines as the types and number of mutations needed to develop the resistant phenotype 79). Therefore, there is a difference in prevalence of the resistance associated mutations that selected from DAA administration among these three non-structural proteins.

RASs at positions 53, 80, 122, 155 and 168 within the NS3 protease region are often associated with virological failure with PIs 80. Mechanically, emergence of RASs to reducing the cure rate of PIs is mainly due to the changes of the conformation of viral proteases, which makes it more difficult for PIs to stably bind to drug targets, ultimately resulting in a decrease in SVR. For example, when simeprevir (TMC435) is used for the treatment of HCV infection, the conformation of R155 is good for facilitating its interaction with the simeprevir crystal structure. Moreover, a salt bridge network structure formed between Q80, R155 and D168 is vital to stabilizing this interaction. Nevertheless, the mutation of R155K caused the salt bridge structure to fail to form, which reduces the stability of interactions of drug and protease. Thus, mutations in R155K, D168A, and Q80K ultimately contribute to the effect of simeprevir escaped by NS3 81.

NS5A inhibitors are an indispensable component of all first-line DAA regimens as they are the class of HCV drugs where resistance is most clinically relevant. With respect to NS5A inhibitors, it works mainly by binding to domain 1 of the NS5A dimer, but the specific mechanism of inhibition still remains unclear. The RAS of M/L28T/V, Q/L30E/H/R/S, L31M/V, H58D and Y93C/H/N are the most frequently in NS5A when the patients are treated with NS5A inhibitors 82.Considering the above, RASs of M28A/G/T/V are most frequently in GT1a prior to drug exposure (4-8% M28T/V), and Y93 variants (Y93C/H/N) of NS5A RASs have the most clinical importance (< 7×),which are found most frequently in GT1b (10%) and GT3 (8-10%) but appear rarely in GT1a (< 1%). However, the Variants at position 93 in GT1a can confers very high level resistance to all class NS5A inhibitors except for pibrentasvir (PIB) (< 7×) 83, 84. The occurrence of RASs leads to a decrease in the affinity of the NS5A inhibitor and NS5A, which finally compromises its effectiveness.

The spectrum of mutations associated with NS5B inhibitors is likewise broad. Similar to HIV therapy, HCV polymerase inhibitors also can be subdivided into two types: nucleosides (NIs) and non-nucleoside inhibitors (NNIs). Because all NISs target the highly conserved active sites of polymerases, these inhibitors tend to be pan-generic 85. In the course of treatment with NIs, the most important compound from this class is sofosbuvir. In pre-clinical assessment of sofosbuvir, a serine to threonine substitution at position 282 of polymerase (S282T) conferred a 10-fold resistance against sofosbuvir 86. The *in vitro* analysis of the S282T variant showed that the affinity of the mutated polymerase for nucleoside analogs was reduced, and this substitution also resulted in a significant loss of replication fitness 87. Non- nucleoside inhibitors bind to allosteric binding sites outside the polymerase active site, and their resistance barriers are lower compare with NIs. Dasabuvir is currently the only approved non-nucleoside inhibitor. For dasabuvir, the common RASs in NS5B are M414T and S556G 88, or A421V and P495L/S 89.

In addition to these common resistance mutations, several prevalence studies have demonstrated that there are still some RASs pre-existing prior to the treatment within the viral population of an infected patient. Although not as common as the RASs merging under drug selective pressure, baseline resistance may also affect the outcome of DAA therapy.

As epidemiological studies have shown that Q80K RAS in NS3 is associated with significantly lower SVR rates for treatment with SMV + peg-IFN-α + RBV^90^, existing as a natural polymorphism mainly in HCV GT1a (20-52%) 91. In particular, the presence of Q80K is an especially challenge in G1a infected patients with cirrhotic, as they treated with SMV + SOF, lower SVR rates of 74% were observed in the presence of Q80K versus 92% in the absence of Q80K 92. Therefore, monitoring of Q80K before treatment is recommended in all subjects with HCV GT1a infected, especially treatment with SMV + peg-IFN-α + RBV, while for therapy regimen of SMV + SOF, baseline resistance testing of Q80K is needed only for cirrhotic patients 93.

Variants at positions 31, 54 and 93 are most common in NS5A, including L31M, Q54H and Y93H 94. For the combination regimen of ASV + DCV, the NS5A variant Y93H was pre-existed in half of the G1b infected patients who went through treatment failure. In contrary to this result, in a UNITY-1 study, despite the higher frequency of NS5A RASs was observed prior to treatment in GT1b compared to GT1a infected patients (16% vs 11%), all subjects with GT1b infection achieved SVR in contrast to only 74% for GT1a 95, 96.Nevertheless, a recent study showed an exciting result that the impact of baseline NS5A RASs on outcome of treatment can be significantly reduced or even completely removed when patients go through a longer duration of treatment with SOF + LDV and addition of RBV^96^. Furthermore, pre-existing RASs in NS5A appeared to have little effect on the outcome of treatment regimen with SOF + VEL, in spite of a high frequency of such variants, 97-100% subjects with baseline resistance in NS5A achieved SVR 97, 98.

Variant L159F in NS5B was detected in 1% of the subjects with GT1 infection and was only observed to have a direct association with accumulated virological failure when patients were treated with SOF + RBV for short durations 99. Unlike low prevalence and limited effect of L159F in GT1, 34% prevalence of RAV L159F was observed in GT1b and was significantly associated with dropped SVR rates of 25% as opposed to 65% in patients without this variant 100. In general, a low prevalence of pre-existing RASs was detected in nucleoside inhibitor based regimens 101.

The prevalence of RASs is different among the major genotypes and subtypes of HCV, and it may due to various resistance barriers of them. For example, the most frequent RAS is Y93H in subjects with HCV GT3 and GT1b infection, but rarely observed in patients with HCV GT1a infection 78. In addition, a protease inhibitor-related R155K-resistant mutation in the NS3 protein requires only one nucleotide change (AGG to AAG) in GT1a HCV infected patients as opposed to the occurrence of R155K RAS requires two nucleotide changes (CGG to AAG) in HCV GT1b infected subjects who initiated therapy with a protease inhibitor. As a result, variant at position 155 is lowering prevalent in HCV G1b infected subjects compared to the G1a infected subjects due to higher resistance barrier 78.

Therefore, it remains necessary to detect the HCV genotype and subtype of a patient. Because a correct HCV genotype/subtype determinate by baseline HCV sequencing can provide critical virological information for the detection of genetic variants that have a potential impact on therapy response, which can guide the selection of the optimal antiviral regimen for patients and decrease frequency of treatment failure.

In addition, encouraging epidemiological studies have demonstrated that the prevalence of RAS is also related to geographical distribution, resulting in some of more frequent mutations in some places. Statistically, the overall prevalence of Q80K is 7.5%, decomposed into19.8% in GT1a, 0.5% in GT1b, and 18.2% in other or unknown GT1 subtypes 102. Furthermore, the prevalence of Q80K among GT1 patients may be geographically various: the prevalence of Q80K was 34% in a North American G1 population and reached more than 40% in subjects who infected HCV GT1.In Europe, Q80K prevalence in GT1 ranged from 0% in Bulgaria to 18.2% in the UK. But the prevalence was various from country to country owing to different Q80K prevalence and ratios of G1a/G1b within the G1a genotype 102.

In summary, the drug resistance of DAA is associated with a variety of factors, and RASs can be detected in all patients with treatment failure. Thus, the detection of RASs is critical for the success of treatment, including the RASs naturally occurring and selected out during therapy. Until now, the detection of drug resistance mainly relies on Sanger automated sequencing and second-generation sequencing (NGS), and NGS is an emerging technology with great potential in data analysis and data integrity 78. Of note, international guidelines recommend that RAS present in >15% of sequences are believed clinically significant and should be taken into serious consideration in the selection of treatment regimen 102, 103.Conversely, in a RAS study based on massively parallel, they reported that presence of baseline RASs even much less than 15% of HCV sequences is also associated with treatment failure in two HCV G1a-infected patients treated with ledipasvir (LED)+ sofosbuvir (SOF) 67, 104-106.

Thus, the drug resistance of DAA is a complex and unavoidable problem, which may result in bad response to antiviral therapy and relapse in HCV infected patients. To date, with the aim of decreasing frequency of RASs, it is useful and common to select different classes DAAs for combination treatment. And a correct determination of HCV genotype and subtype and detection of pre-existing RASs in sequence remains important for guiding selection of most appropriate antiviral regimen.

## Conclusion

The combination, oral DAA (±RBV) therapies have become the clinical treatment of choice, which has revolutionized cure rate for patients with HCV infection. In addition to the excellent antiviral effect that more than 91% SVR can be achieved in patients, there are some unexpected effects of recovery in liver dysfunction, metabolic disorder and immune function damage in patients with clearance of HCV compared to IFN-based therapy. However, presence of baseline resistance and RASs occurring during antiviral treatment is critical and notable a challenge, which can be observed in all patients with treatment failure. Therefore, with the aim of reduction of frequency in RASs and non-responders, selection of DAA therapies should be taken with many factors into consideration, including correct determination of HCV genotype and subtype and detection of RAS in sequence. Thus, the exploitation of antiviral drugs suitable for the all main HCV genotypes, to minimize the incidence of resistance mutations in order to further improve the sustained viral response, remains a focus of future research and with a great pool of knowledge based on profound researches, more effective therapies are hoped to emerge.

## Figures and Tables

**Figure 1:**
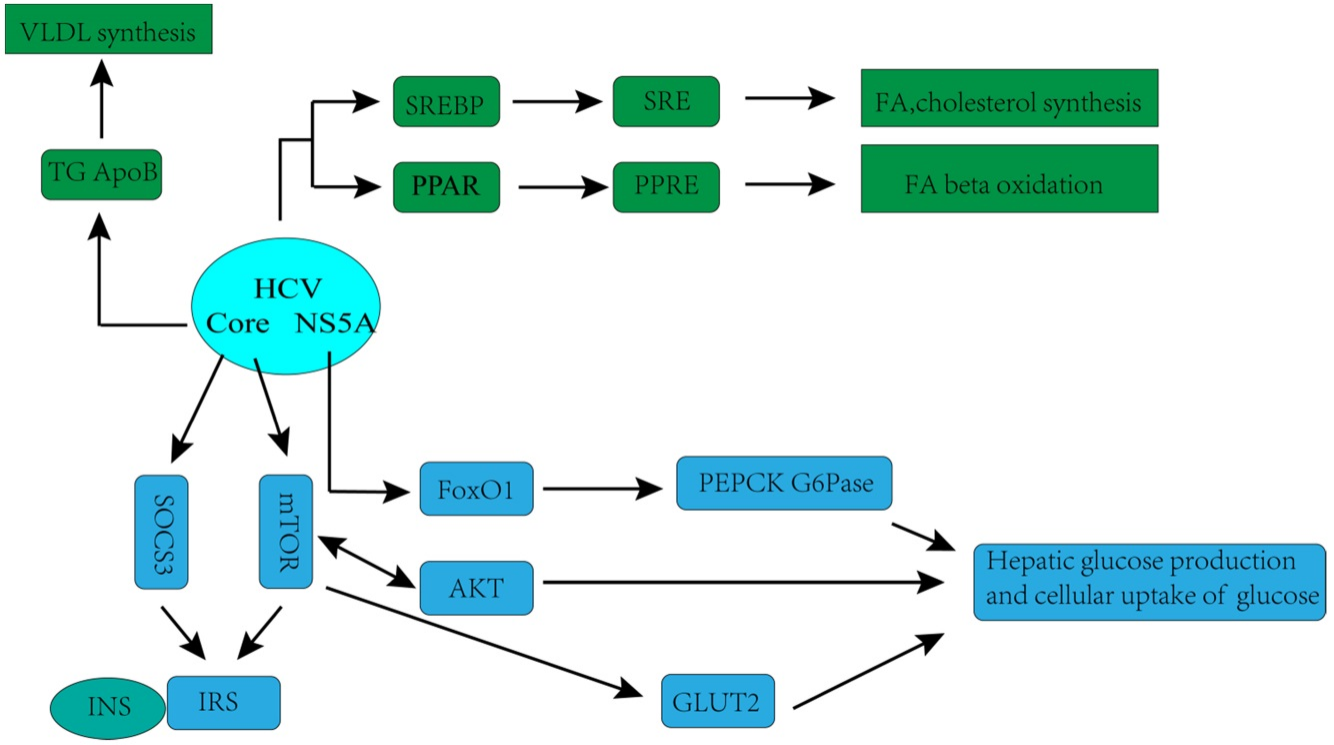
** Hepatitis C virus (HCV)-associated glucose and lipid metabolic changes 32.** GLUT2, glucose transporter 2; INS, insulin; IRS, insulin receptor substrate; mTOR, mammalian target of rapamycin; SOCS3, suppressor of cytokine-signalling protein; AKT, protein kinase B (Akt); FoxO1,forkhead box protein O1, transcription factor; PEPCK, phosphoenolpyruvate carboxykinase; G6Pase, glucose-6-phosphatase; SREBP, sterol regulatory element-binding protein; SRE, sterol regulatory element; PPAR, peroxisome proliferator-activated receptor; PPRE, PPAR response element; NS5A, HCV non-structural protein 5A;VLDL, very-low-density lipoprotein cholesterol; ApoB, apolipoprotein B; TG, triglycerides; FA, fatty acid.

**Figure 2:**
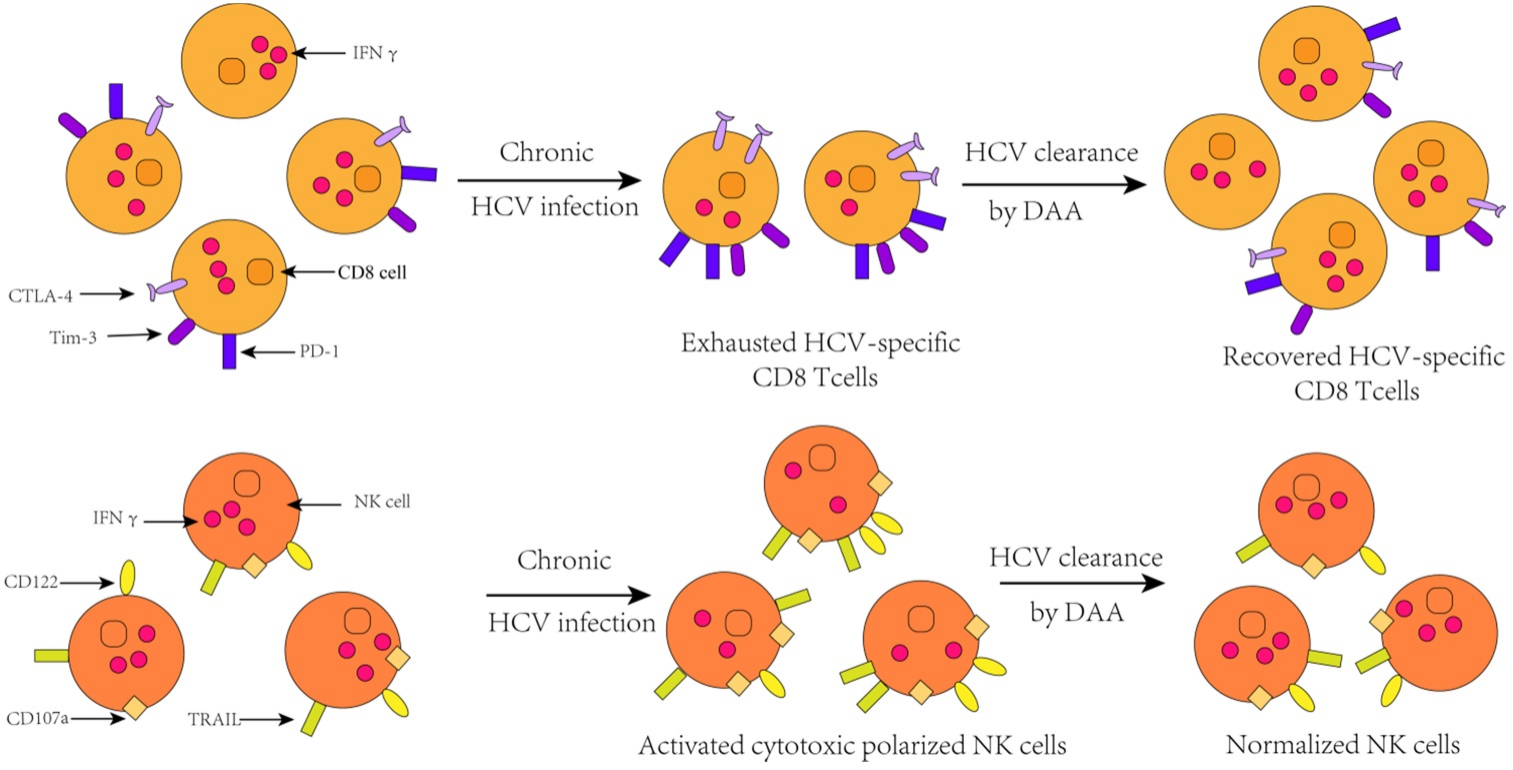
Change of immune cell function after HCV clearance by successful DAA treatment^76^.

**Table 1:** Cross-resistance pattern of clinically used NS3, NS5A, and NS5b inhibitors (2-fold to>100-fold change resistance)^78^.

Category	Name	Resistance-associated substitutions
NS3/4 protease inhibitors (PIs)	asunaprevir, simeprevir, paritaprevir, grazoprevir	F53S, Q80K/R, S122R, R155K, A156T/V, D168 any
NS5A inhibitors	daclatasvir, ledipasvir, ombitasvir, elbasvir, Pibrentasvir	M28A/G/T, Q30E/H/R, L31F/M/V, P32L/S, H58D, Y93H
NS5B inhibitors	sofosbuvir, dasabuvir	C316N
